# Repeated Mandibular Extension in Rat: A Procedure to Modulate the Cerebral Arteriolar Tone

**DOI:** 10.3389/fphys.2017.00625

**Published:** 2017-08-31

**Authors:** Dominga Lapi, Maurizio Varanini, Antonio Colantuoni, Cristina Del Seppia, Sergio Ghione, Enza Fommei, Rossana Scuri

**Affiliations:** ^1^Department of Clinical Medicine and Surgery, School of Medicine, University of Naples Federico II Naples, Italy; ^2^Institute of Clinical Physiology, National Council of Research Pisa, Italy; ^3^Medical and Public Health Research, Fondazione Toscana Gabriele Monasterio (CNR) Pisa, Italy; ^4^Department of Translational Research on New Technologies in Medicine and Surgery, University of Pisa Pisa, Italy

**Keywords:** arteriolar diameter changes, blood pressure, endothelial activity, heart rate, mandibular extension, pial microcirculation

## Abstract

Previous data have shown both in the rat and in the human that a single mandibular extension lasting 10 min induces a significant important and prolonged reduction in blood pressure and heart rate, affecting also rat pial microcirculation by the release of endothelial factors. In the present work, we assessed whether repeated mandibular extension could further prolong these effects. We performed two mandibular extensions, the second mandibular extension being applied 10 min after the first one. The second mandibular extension produced a reduction in blood pressure and heart rate for at least 240 min. As in the case of a single mandibular extension, pial arterioles dilated persisting up to 140 min after the second extension. Spectral analysis on 30 min recordings under baseline conditions and after repetitive mandibular extensions showed that the pial arterioles dilation was associated with rhythmic diameter changes sustained by an increase in the frequency components related to endothelial, neurogenic, and myogenic activity while a single mandibular extension caused, conversely, an increase only in the endothelial activity. In conclusion, repetitive mandibular extension prolonged the effects of a single mandibular extension on blood pressure, heart rate and vasodilation and induced a modulation of different frequency components responsible of the pial arteriolar tone, in particular increasing the endothelial activity.

## Introduction

A single mandibular extension (ME), consisting in a submaximal mouth opening for 10 min induced with an ad hoc dilator, has been shown to cause an unusually prolonged reduction (of almost 3 h) of heart rate (HR) and mean arterial blood pressure (MABP) (Lapi et al., 2013, 2014) in normotensive anesthetized rats. Similar results have been detected in young volunteers (Brunelli et al., 2012). In the rat, the hypotensive and bradycardic effects have been found to be associated to a characteristic biphasic response of the pial arteriolar diameters, consisting in an initial brief vasoconstriction concomitant with ME followed by a prolonged vasodilation lasting for the whole experimental observation period of 3 h. Although, many aspects related to the underlying mechanisms remain to be established, some of them have been clarified. The effects produced by ME are abolished by cutting the peripheral trigeminal branches, indicating that the hypotensive and bradycardic effects may be ascribed to the so-called trigemino-cardiac reflex (Lapi et al., 2013). Moreover, the effects are depend on the ME duration, 10 min ME resulting the optimal stimulus (Lapi et al., 2013). The initial brief vasoconstrictory response involves an opioid receptor-mediated mechanism, and the subsequent prolonged vasodilation is related to NO intracellular signaling (Lapi et al., 2014).

Recently, by expanding the number of normotensive humans studied, Del Seppia and coworkers (Del Seppia et al., 2016, 2017) have confirmed the mild but prolonged blood pressure and heart rate lowering effect after a 10 min mandibular extension. These effects were induced by a submaximal mouth opening associated with partial masticatory movements or by a fixed mouth opener. The prolonged reduction in blood pressure without evidence of baroreflex-mediated compensatory tachycardia suggests that mandibular extension may induce a prolonged resetting of the operating point of the arterial baroreflex to a lower pressure (Minami et al., 2006).

Therefore, further studies are required to ascertain whether the hypotensive response could be improved by optimizing the different components of mandibular extension (i.e., method used, duration, intensity, and repetition) because although the blood pressure decrements observed were modest they are in the same order of magnitude (5–10 mmHg) of those reported for other non-pharmacological treatments proposed for the control of arterial hypertension. Moreover, the mandibular extension might represent a procedure useful in the control of the systemic blood pressure and consequently of the organs perfusion, in particular of the brain being highly susceptible to the blood pressure variations.

Therefore, the aim of the present study was twofold: on one hand to clarify if the cardiovascular effects could be prolonged when two mandibular extensions were applied; on the other hand to analyze the rhythmic oscillations of the rat pial arterioles diameter after ME because the changes of the smaller arterioles tone are the basis of the blood flow regulation to the tissue. In particular, the diameter changes were analyzed with an appropriate power spectrum analysis technique.

In fact the arterioles display rhythmic variations in diameter, initiated by smooth muscle cells and sustained by the endothelium that plays a modulatory role on vessel tone (Aalkjaer and Nilsson, 2005; Haddock and Hill, 2005). Several studies have been carried out to assess the components of this vasomotor activity and the molecules affecting the arteriolar diameter changes (Funk et al., 1983; Bertuglia et al., 1991, 1999; Bollinger et al., 1991; Achakri et al., 1994; Colantuoni et al., 1994; Kvandal et al., 2003; Nilsson and Aalkjaer, 2003; Krupatkin, 2008; Tankanag and Chemeris, 2008). Six main components were identified by Stefanowska and coworkers (Stefanovska et al., 1999; Kvandal et al., 2006) in the arm skin circulation using a laser Doppler technique on prolonged recordings. Therefore, rat pial arteriolar diameter changes were studied on 30 min recordings.

## Materials and methods

### Animals

**Figure 1:**
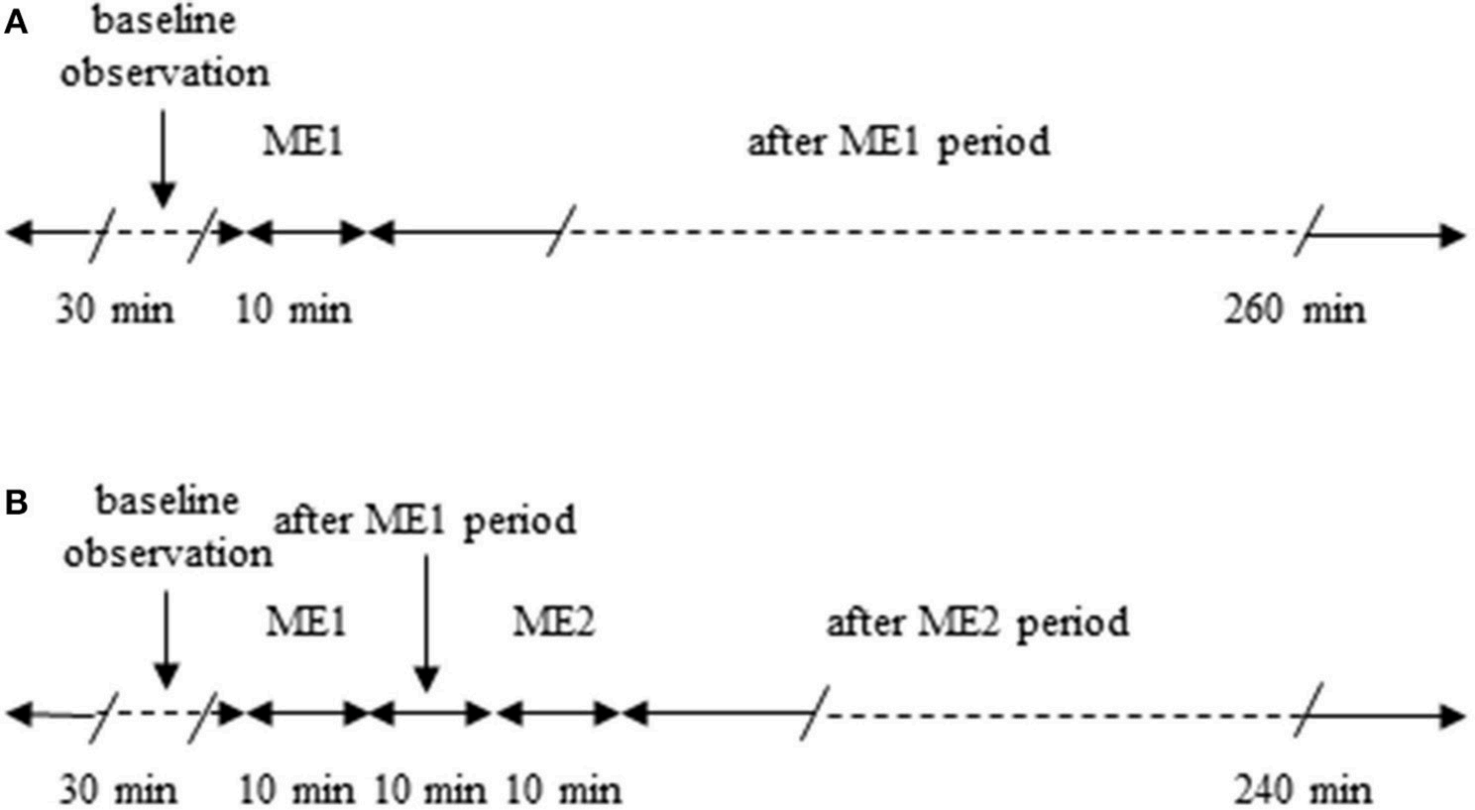
Outline of the protocols used. **(A)** Single ME protocol: after 30 min of observation in baseline conditions, 10 min ME (ME1) was performed and after removing ME post-treatment measurements were carried out for 240 min. **(B)** Early repetition protocol: after 30 min of baseline observation, 10 min ME (ME1) were performed and after 10 min from removing ME1 further 10 min mandibular extension (ME2) were applied; then after removing ME2 post-treatment measurements were carried out for 240 min.

Male Wistar rats (250–300 g), (Harlan, Udine, Italy) were randomly assigned to one of the following groups: (i) rats subjected to a single 10 min ME (*n* = 7) and monitored for 260 min after ME (Figure 1A); (ii) rats (*n* = 7) submitted to two 10 min ME. The second ME (ME2) was applied 10 min after the first one (early repetition), as described in Figure 1B and monitored for 240 min after ME2; (iii) rats subjected to a topical administration 10 min prior to ME of N-iminoethyl-L-ornithine (L-NIO: 100 μM), inhibitor of nitric oxide synthase (*n* = 3; Saleron et al., 2002) or of charybdotoxin (100 nM) plus apamin (10 nM) inhibitors of endothelium-derived hyperpolarizing factor (*n* = 3) and submitted to a single ME; (iv) rats subjected to a topical administration 10 min prior to ME1 of L-NIO (100 μM), (*n* = 3), or of charybdotoxin (100 nM) plus apamin (10 nM) (*n* = 3) and submitted to the early repetition protocol; (v) rats subjected only to the surgical procedures without ME (sham operated, SO, *n* = 5) or rats SO subjected to topical administration of L-NIO (100 μM) (*n* = 3) or rats SO subjected to topical administration of charybdotoxin (100 nM) plus apamin (10 nM) (*n* = 3) and monitored for 300 min.

This study was carried out in accordance with the recommendations in the Guide for the Care and Use of Laboratory Animals of the National Institute of Health. The protocol was approved by the Committee on the Ethics of Animal Experiments of the University of Pisa (Permit Number: 00 4896/2013). All surgery was performed under alpha-chloralose and urethane anesthesia and all efforts were made to minimize suffering.

### Animal preparation

The rats were anesthetized with intraperitoneal injection of alpha-chloralose (50 mg/Kg b.w.) (Sigma-Aldrich, St. Louis, MO, USA) plus urethane (600 mg/Kg b.w.) (Sigma-Aldrich, St. Louis, MO, USA) for induction and with urethane alone (100 mg/kg, i.v., every hour) for maintenance. They were tracheotomized, intubated and mechanically ventilated with room air and a supplemental mixture of O_2_/CO_2_ (concentration of O_2_ = 95%, CO_2_ = 5%); the end-tidal CO_2_ was continuously measured by a CO_2_ analyzer and a respirator was adjusted to maintain end-tidal CO_2_ from 4.5 to 5.0% and to keep arterial blood gas tension within the normal range (34.0 ± 2.5 mmHg for PaCO_2_ and 90 ± 4 mmHg for PaO_2_). A catheter was placed in the left femoral artery, that allowed to measure the arterial blood pressure and to draw arterial blood samples at 60-min intervals for monitoring PaO_2_, PaCO_2_, and pH using a blood gas/pH analyzer (ABL 80FLEX ANALYZER, RADIOMETER). pH, PaCO_2_, and PaO_2_ were maintained within physiological ranges (7.4 ± 0.01 for arterial pH, 35 ± 4 mmHg for PaCO_2_ and 90 ± 5 mmHg for PaO_2_) throughout the study. Another catheter was placed in the right femoral vein and used for the injection of the fluorescent tracer fluoroisotiocyanate (FITC) (Sigma-Aldrich, St. Louis, MO, USA) about every 120 min and of urethane, about every 60 min, during the whole experiment.

Rectal temperature was monitored and maintained at 37.0 ± 0.5°C with a heating stereotaxic frame, where the rats were secured.

To observe the pial microcirculation, a closed cranial window (4 × 5 mm) was implanted above the left parietal cortex (posterior 1.5 mm to bregma; lateral, 3 mm to the midline; Lapi et al., 2008). To prevent overheating of the cerebral cortex during drilling, cold saline solution was suffused on the skull. The dura mater was gently removed and a 150 μm-thick quartz microscope coverglass was sealed to the bone with dental cement. The window inflow and outflow were assured by two needles secured in the dental cement of the window so that the brain parenchyma was continuously superfused with artificial cerebrospinal fluid (aCSF). The rate of superfusion was 0.5 mL/min and was controlled by a peristaltic pump. During superfusion the intracranial pressure was maintained at 5 ± 1 mmHg and measured by a pressure transducer connected to a computer.

### MABP and HR measurements

Throughout all experiments MABP and HR were continuously recorded. MABP was monitored with a blood pressure transducer BLPR2 (World Precision Instruments, Sarasota, FL, USA) connected to an ad hoc bridge amplifier (home-made) and recorded using a LabView software (National Instruments S.R.L., Milan, Italy). ECG recording (at a sampling rate of 1 KHz) was done with a home-made differential amplifier for biomedical signals and data acquisition was obtained by a LabView software. HR was calculated from the R-R interval. Data were recorded and stored in a computer for off-line analyses.

The average of three measurements obtained every 10 min over the 30 min immediately prior to ME1 (baseline observation) represented the baseline value. Post-treatment measurements were obtained immediately after ME and every 5 min for the whole observation period. For a better graphical representation, in the graphs data were plotted every 20 min.

### Mandibular extension

Mandibular extension (ME) was induced as previously described (Lapi et al., 2014). Briefly, an appropriately U-shaped spring device was placed between the superior and inferior dental arches of the rat. The spring device consisted of two thin layers covered with a silicone elastomer (Sylgard, Dow Corning, Midland, MI) coupled to an adjustable spring, allowing to open the month without muscle fatigue.

### Fluorescent microscopy technique and microvascular parameter assessment

Pial microcirculation was visualized with a fluorescence microscope (Leitz Orthoplan) fitted with long-distance objectives 2.5 x, numerical aperture (NA) 0.08, 10 x, NA 0.20, 20 x, NA 0.25, 32 x, NA 0.40, and a 10 x eyepiece and a filter block (Ploemopak, Leitz). Epi-illumination was provided by a 100 W mercury lamp using the appropriate filter for FITC and a heat filter (Leitz KG1). Pial microcirculation was televised with a DAGE MTI 300RC low-light level digital camera (2.1 megapixel) and recorded by a computer based frame grabber (Pinnacle DC 10 plus, Avid Technology, MA, USA).

Video images were continuously videotaped and microvascular measurements (diameter and length) were made off-line using a computer-assisted imaging software system (MIP Image, CNR, Institute of Clinical Physiology, Pisa, Italy). The results of diameter measurements were in accordance with those obtained by the shearing method (±0.5 μm). To avoid bias due to single operator measurements, two independent “blinded” operators measured the vessel diameters.

The arteriolar network was mapped by stop-frame images and pial arterioles were classified according to a centripetal ordering scheme (Strahler method, modified according to diameter) (Kassab et al., 1993; Lapi et al., 2008).

In each animal one order 5, one order 4, two order 3, and four order 2 arterioles were identified. In this paper we present the data of order 2 vessels, that represent the main site of regulation of blood supply to capillary network (Pradhan and Chakravarthy, 2011).

Video images were continuously videotaped and the diameter measures of pial arterioles were obtained on 1 min tracings every 10 min (acquisition frequency: 4 Hz).

In sham operated rats (not subjected to ME), all parameters were monitored and recorded for 300 min.

**Figure 2:**
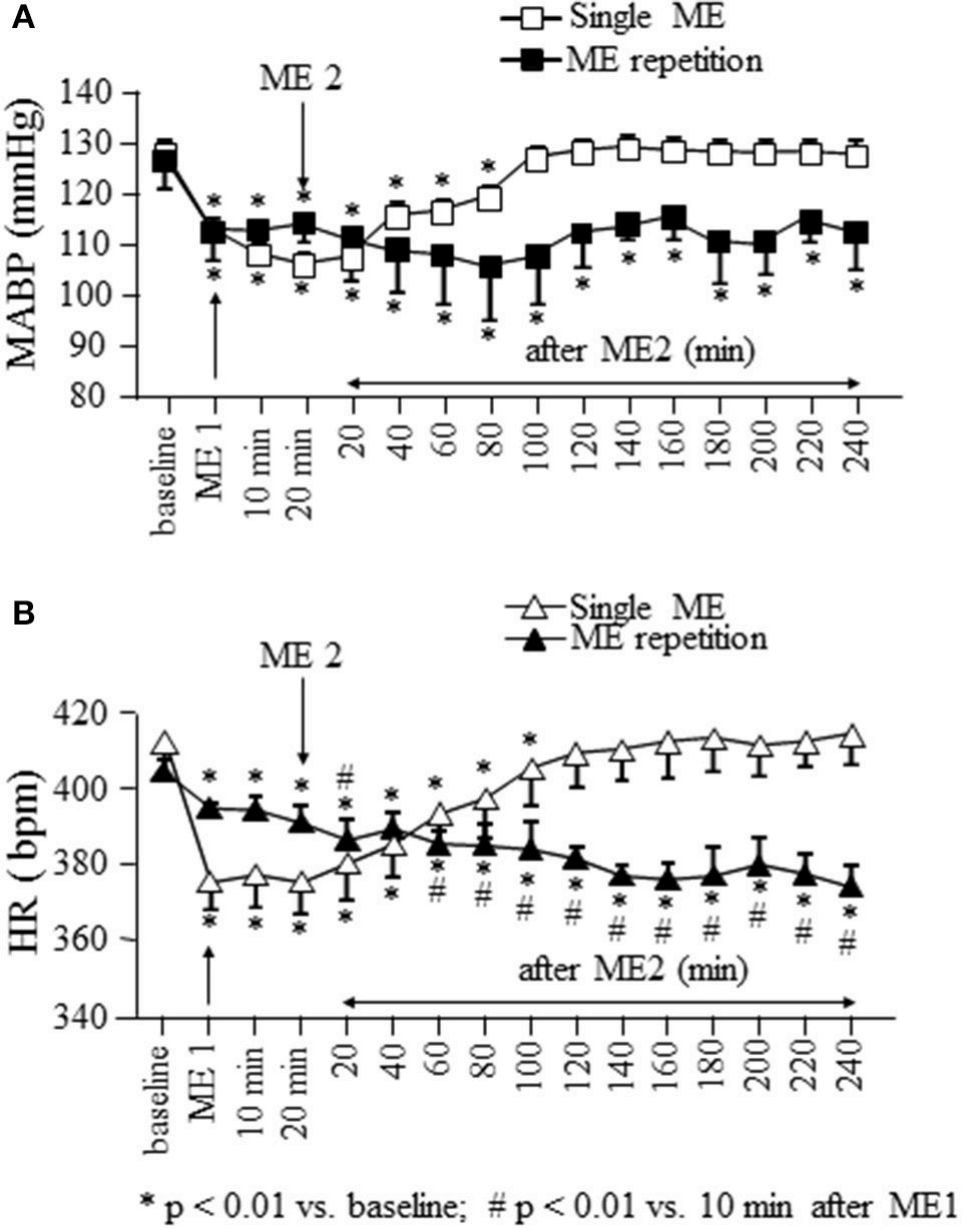
Effects of a single ME and early ME repetition on MABP and HR. Rats subjected to a single ME, exhibited a reduction in MABP (□ in **A**) and HR (△ in **B**) which persisted only for 100 and 120 min, respectively. In the early ME repetition protocol, ME1 caused a significant reduction of MABP (■ in **A**) and HR (▴ in **B**) compared with the baseline values. The application of ME2 10 min after removing ME1 maintained MABP reduced for the whole observation period, while produced a further decrement of HR which persisted for the whole observation period. ^*^Indicates significantly difference from the baseline value; #Indicates significantly difference from the values observed after ME1.

**Figure 3:**
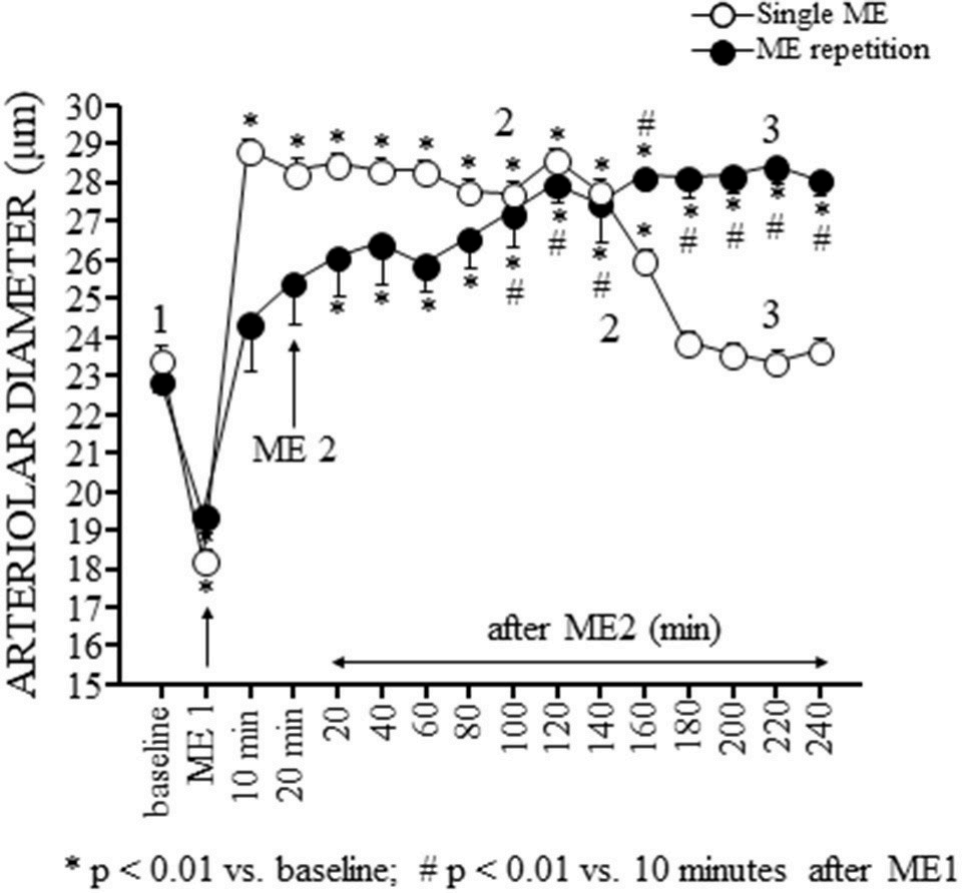
Effects of a single ME and early ME repetition on pial arterioles. The values of the diameters of order 2 arterioles are reported. In the rats subjected to a single ME (○), the initial vasoconstriction was followed by a vasodilation that persisted only for 180 min. In the early ME repetition protocol, ME1 induced an initial brief vasoconstriction followed by a vasodilatation of pial arterioles that gradually increased after ME2 up to 160 min. Subsequently, the pial arterioles remained dilated for the entire observation period. The numbers 1, 2, and 3 plotted in the figure indicate the time intervals in which the frames for the spectral analysis showed in Figures 4, 5 has been recorded. ^*^Indicates significantly difference from the baseline value; #Indicates significantly difference from the values observed after ME1.

**Figure 4:**
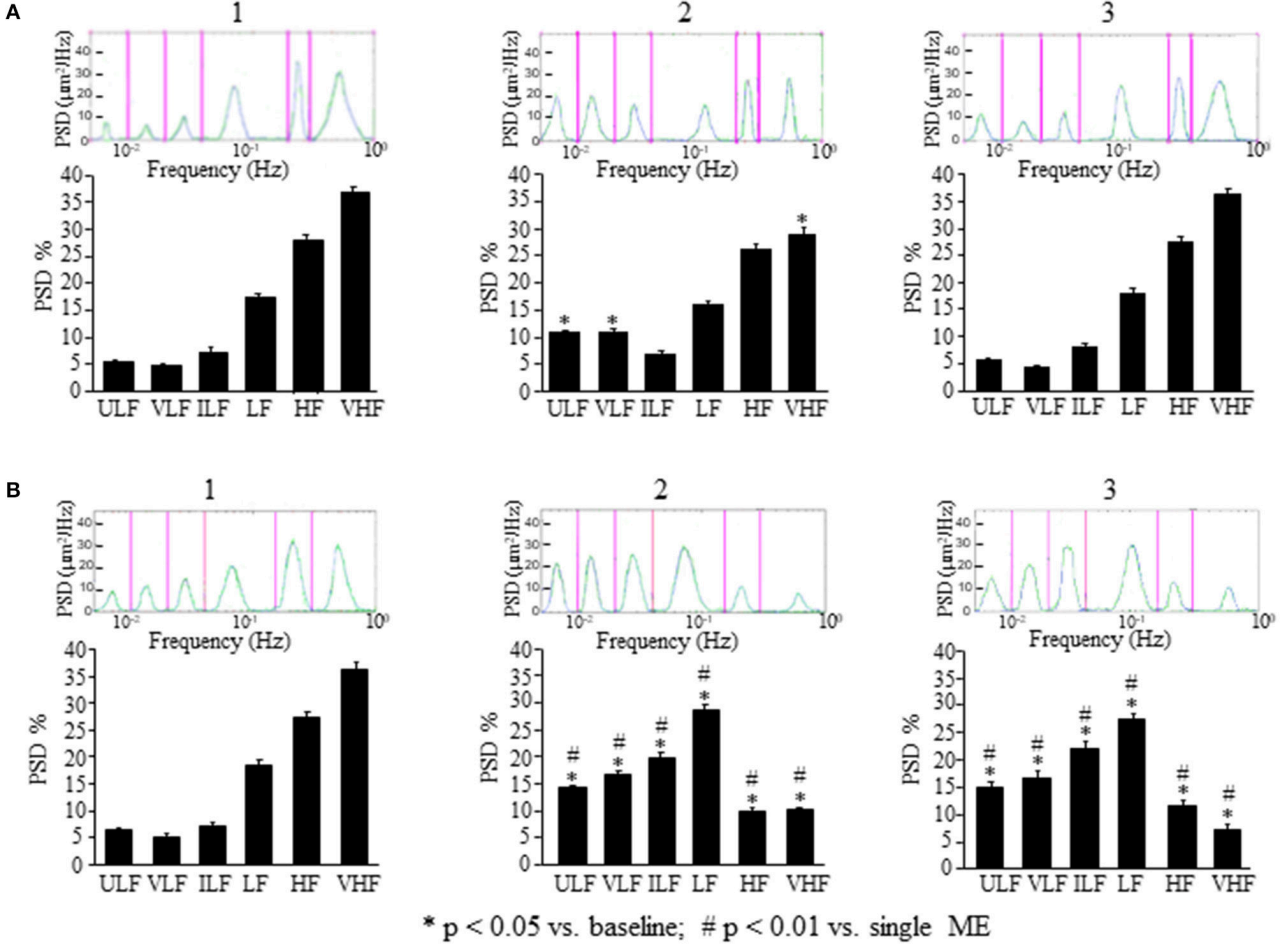
Comparison between the effects of a single ME or of early ME repetition on rhythmic diameter changes. **(A)** In the rats subjected to a single ME, the rhythmic diameter changes (upon) and the main corresponding frequency components (bottom), expressed as percent normalized power spectral density (PSD: μm^2^/Hz) were measured in baseline conditions (1), at the pick of the vasodilation with recordings between 120 and 150 min (2), and during the recovery of the baseline conditions with recordings between 230 and 260 min (3). ME caused a significant increase of the ULF and VLF frequency components, and a decrease of VHF component **(A2)** compared with the baseline conditions **(A1)**; afterwards all frequency components recovered the baseline values **(A3)**. **(B)** In the rats subjected to early ME repetition, the rhythmic diameter changes (upon) and the main corresponding frequency components (bottom), expressed as percent normalized power spectral density (PSD: μm^2^/Hz) were measured in baseline conditions (1), between 120 and 150 min after ME2 (2), and between 210 and 240 min after ME2 (3). ME2 caused a significant increase in ULF, VLF, ILF, and LF spectral density and a reduction in HF and VHF spectral density **(B2)** compared with the baseline conditions **(B1)** and A2 conditions (# in the diagrams) These effects persisted for the whole observation period **(B3)**, maintaining significantly different respect to A3 conditions (# in the diagrams). ULF, ultra-low frequency component; VLF, very low frequency component; ILF, intermediate frequency components; LF, low frequency component; HF, high frequency component; VHF, very high frequency component. ^*^Significantly different from the baseline value.

**Figure 5:**
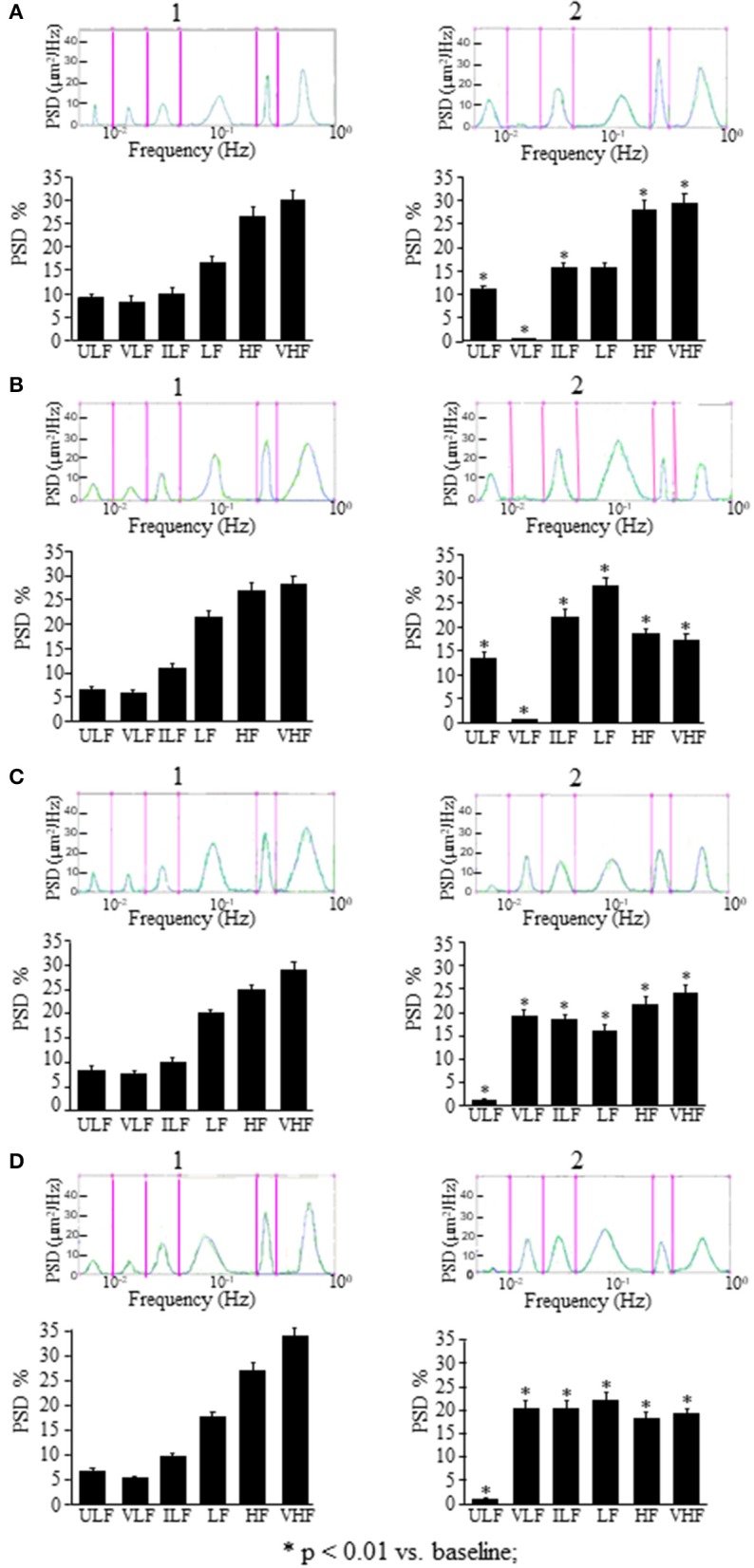
Effects of NOS inhibition or EDHF inhibition on rhythmic diameter changes after a single ME or early ME repetition. **(A)** In the rats subjected to a single ME, the rhythmic diameter changes (upon) and the main corresponding frequency components (bottom), expressed as percent normalized power spectral density (PSD: μm^2^/Hz) show that NOS inhibition abolished the VLF frequency components after ME **(A2)** respect to the baseline conditions **(A1)**, and EDHF inhibition abolished the ULF frequency components **(C2)** respect to the baseline conditions **(C1)**. The measures were obtained on recordings carried out 120–150 min after ME. Also after early ME repetition, NOS inhibition abolished the VLF frequency components after ME2 **(B2)** respect to the baseline conditions **(B1)**, while; EDHF inhibition abolished the ULF frequency components after ME2 **(D2)** respect to the baseline conditions **(D1)**. The measures were obtained on recordings carried out 120–150 min after ME2. ULF, ultra-low frequency component; VLF, very low frequency component; ILF, intermediate frequency components; LF, low frequency component; HF, high frequency component; VHF, very high frequency component. ^*^Significantly different from the baseline value.

Baseline measurements were obtained prior to ME1 (average of three measurements at 10 min intervals over the 30 min immediately prior ME1) and defined as baseline values (baseline in the Figures 2–**5**). Post-treatment measurements were obtained immediately after ME1 or ME2 (ME1 or ME2 in the Figures 2–**5**), 10 and 20 min after ME1 in the single ME protocol, 10 min after ME1 in the early repetition protocol and were continued every 20 min up to the end of the experiment.

### Evaluation of rhythmic diameter changes

The rhythmic variations in diameter of pial arterioles were analyzed with a computer-assisted power spectrum method, based on the generalized short time Fourier transform (GSTFT) (Varanini et al., 1998; Pradhan and Chakravarthy, 2011; Varanini, 2011), a multiresolution transform which allowed us to choose, at each frequency, the most appropriate balance between time and frequency resolution according to the user's requirements. A Hamming window was used and spectra were computed at frequencies spaced proportionally to the frequency resolution. The power density spectral distribution was obtained by time averaging the time-frequency power density representation. This technique permits to evaluate non stationary data, such as those represented by rhythmic variations in vessel diameters (Rossi et al., 2005).

The diameter measures used for the analyses were obtained on 30 min tracings (acquisition frequency: 4 Hz) to obtain the resolution of the six components in the following ranges: 2.5–4.5 Hz (termed VHF, i.e., very high frequency), 0.2–2.0 Hz (HF, high frequency), 0.06–0.2 Hz (LF, low frequency),0.02–0.06 Hz (ILF, infra-low frequency), 0.0095–0.021 Hz (VLF, very low frequency), and 0.001–0.0095 Hz (ULF, ultra-low frequency; Lapi et al., 2010).

The visualization of pial microcirculation was obtained by infusion of FITC. Because FITC enters the bloodstream after 5 min from the administration in the femoral vein and it persists in the circulation for about 60 min, we provided FITC every 60 min for the whole duration of the experiments.

In the animals subjected to a single ME, the analysis was carried out on frames recorded in baseline conditions, between 120 and 150 min and between 230 and 260 min after ME, while in the rats subjected to early repetition protocol the analysis was performed in baseline conditions and between 110 and 140 min after ME2.

### Statistical analysis

All data were expressed as mean ± SE. Data were tested for normal distribution with the Kolmogorov–Smirnov test. Due to the normality of distribution, one way ANOVA for repeated measures was used for testing the changes in MABP, HR, and pial arterioles diameter respect to the baseline values. For *post-hoc* analysis, the Dunnett's multiple comparison test was done.

The results obtained in the spectral analysis were analyzed by Wilcoxon test and Man–Whitney test because the data did not pass Kolmogorov–Smirnov test.

The statistical analysis was carried out by GraphPad Prism statistical package (version 4.0, Graph Pad Software Inc., San Diego, CA). Statistical significance was set at *p* < 0.05.

## Results

### Effects of a single ME on MABP, HR, and pial microcirculation

In rats subjected to a single ME and monitored for 260 min after removing of mandibular extension, MABP (gray square in Figure 2A) showed a significant reduction from 128.2 ± 2.3 to 103.3 ± 2.0 mmHg after ME [*F*_(15, 47)_ = 81.45, *p* < 0.0001] and remained significantly reduced [109.5 ± 1.9 mmHg, *p* < 0.01 vs. baseline) up to 100 min, corresponding to 80 min after ME2 in Figure 2A. Heart rate (gray triangles in Figure 2B) showed a significant reduction from 412.0 ± 10.3 to 375.0 ± 7.2 bpm after ME [*F*_(15, 47)_ = 92.20, *p* < 0.0001] recovering the baseline values (409.0 ± 8.9 bpm, *p* > 0.05) starting from 120 min, corresponding to 100 min after ME2 in Figure 2B.

Pial arterioles diameter (white circles in Figure 3) reduced during ME afterward increased. The progressive dilation resulted statistically significant up to 180 min, corresponding to 160 min after ME2 in Figure 3, then the diameter recovered the baseline value.

The analysis of the rhythmic oscillations of the diameters carried out on 30 min recordings between 120 and 150 min after ME (corresponding to 100 and 130 min after ME2 in Figures 3, 4A2) showed a significant increase in spectral density of ULF (*p* < 0.001) and VLF (*p* < 0.001) frequency components and a decrease of VHF component (*p* < 0.05) compared with the baseline conditions (Figure 4A1), and in the recordings performed between 230 and 260 min after ME (corresponding to 210 and 240 min after ME2 in Figure 3) all frequency components recovered the baseline values (Figure 4A3).

In the rats treated with L-NIO, the spectral analysis carried out on recordings acquired between 120 and 150 min after ME (Figure 5A2) showed some significant changes of the frequency components: ULF was significantly increased [*F*_(2, 14)_ = 72.06, *p* < 0.01 vs. baseline] while VLF was abolished compared with the baseline conditions (Figure 5A1); ILF and HF frequency components significantly increased compared with the baseline conditions [*F*_(2, 14)_ = 37.09, *p* < 0.01; *F*_(2, 14)_ = 371, *p* < 0.01 vs. baseline, respectively] and VHF frequency component significantly decreased [*F*_(2, 14)_ = 108, *p* < 0.01 vs. baseline] while LF frequency component did not change (Figure 5A2). These effects persisted for the whole post-ME period (data not shown).

In the rats treated with charybdotoxin plus apamin, in the recordings between 120 and 150 min after ME (Figure 5C2) ULF frequency component was abolished [*F*_(2, 14)_ = 4.923, *p* < 0.01], VLF and ILF frequency components significantly increased [*F*_(2, 14)_ = 17.29, *p* < 0.01 and *F*_(2, 14)_ = 963.6, *p* < 0.01; respectively), while LF, HF, and VHF frequency components significantly decreased [*F*_(2, 14)_ = 97.32, *p* < 0.01, *F*_(2, 14)_ = 26.27, *p* < 0.01 and *F*_(2, 14)_ = 186.7, *p* < 0.01; respectively) compared with the baseline conditions (Figure 5C1). Similar significant changes respect to the baseline conditions persisted in all frequency components for the whole recording period (data not shown).

### Effects of early ME repetition on MABP, HR, and pial microcirculation

Figure 2 shows also the time course of MABP and HR when a second ME (ME2) was applied 10 min after the first ME (ME1). During ME1 the rats exhibited a significant reduction in MABP (black squares in Figure 2A) and HR (black triangles in Figure 2B) from: 127 ± 7 to 113 ± 7 mmHg [*F*_(15.47)_ = 2.989, *p* < 0.0001, *post-hoc* test *p* < 0.05 vs. baseline] and from 404.0 ± 3.5 to 394.0 ± 1.3 bpm [*F*(15.47) = 53.82, *p* < 0.0001, *post-hoc* test *p* < 0.01 vs. baseline), respectively.

ME2 did not produce a further decrease in MABP respect to that observed after ME1, but prolonged the hypotensive effect for the entire observation period (240 min, MABP: 113 ± 7 mmHg; *p* < 0.01 vs. baseline). Conversely, HR decreased further (386.0 ± 5.3 bpm; *p* < 0.01 vs. after ME1) within 20 min from ME2 and persisted reduced for the entire after ME2 period (at 240 min 373.5 ± 5.7 bpm; *p* < 0.01 vs. baseline and 10 min after ME1; Figure 2B).

The pial arteriolar diameter (black circles in Figure 3) decreased during ME1; afterward gradually increased and this rise resulted significant compared to baseline value (^*^ in Figure 3) starting from 20 min after ME2 and respect to the value measured 10 min after ME1 (# in Figure 3) starting from 100 min after ME2. At 120 min after ME2 the dilation reached a plateau that lasted afterward up to the end of the observation period.

This vasodilation was associated with changes in the rhythmic oscillations of diameters (Figure 4B). The spectral analysis of diameter changes performed on recordings carried out between 120 and 150 min after ME2 (Figure 4B2), showed a significant increase of the components related to endothelial activity (ULF, Wilcoxon test, *p* < 0.05 and VLF, Wilcoxon test, *p* < 0.05), neurogenic (ILF, Wilcoxon test, *p* < 0.05) and myogenic activity (LF, Wilcoxon test, *p* < 0.05) respect to the baseline conditions and respect to 120–150 min after the single ME (Figure 4B1). HF and VHF components, on the contrary, showed a significant decrease after ME2 (Wilcoxon test, *p* < 0.05 and *p* < 0.05, respectively) compared both with the baseline conditions and the 120–150 min after the single ME (Figure 4B2, Mann–Whitney, *p* < 0.0001 in both cases). In the recordings performed between 210 and 240 min after ME2 all frequency components maintained the same values detected between 120 and 150 min (Figure 4B3).

In the rats treated with L-NIO (Figure 5B) after ME2, VLF frequency component (Wilcoxon test *p* < 0.05) was abolished, ULF, ILF, and LF frequency components (Wilcoxon test *p* < 0.05) were increased, and HF and VHF frequency components (Wilcoxon test *p* < 0.05) were reduced (Figure 5B2) compared with the baseline conditions (Figure 5B1).

In the rats treated with charybdotoxin plus apamin (Figure 5D), after ME2, ULF frequency component was abolished (Wilcoxon test *p* < 0.05), VLF, ILF, and HF frequency components significantly increased (Wilcoxon test *p* < 0.05), and LF and VHF frequency components significantly reduced (Wilcoxon test *p* < 0.05; Figure 5D2) respect to the baseline conditions (Figure 5D1).

No statistically significant changes in MABP, HR, and arteriolar diameters were detected for 300 min in SO rats (data not shown). Topical application of all substances did not affect MABP and HR for the whole observation period (data not shown).

## Discussion

Previous data, in normotensive rats, demonstrated that a single brief (10 min) and passive ME caused a significant and prolonged decrease of MABP and HR and, in pial arterioles, a decrease of the diameter concomitant to ME followed by an increase after removing ME. Interestingly, these arteriolar responses have been found to be mediated by different mechanisms (Lapi et al., 2013, 2014).

In the present paper, we show that this vasodilation, due to NO release by endothelial vascular cells as previously described (Lapi et al., 2014), is accompanied by changes in spontaneous oscillations of the arteriolar diameters, as assessed by spectral analysis, consisting in an increase of the two lowest frequency components likely to be related to the endothelial activity (ULF and VLF), with concomitant reduction of the highest frequency component (VHF) (Figure 4A).

Interestingly, the effects on pial arterioles as well as on MABP and HR were even more prolonged when a second ME (ME2) was applied 10 min after the first ME (early repetition). With regards to the vessels rhythmic diameter changes, spectral analysis showed that ME repetition produced a further increase of the ULF and VLF components compared with those observed after a single ME (compare Figure 4A2 and Figure 4B2). Pial arterioles exhibited a further dilation 80 min after ME2, compared with the baseline value and the values observed after a single ME. This pronounced vasodilation lasted for 240 min and was accompanied by a significant increase not only of the endothelial-related frequency components (ULF and VLF) but also of the neurogenic and myogenic activity-related components (ILF and LF; Figure 4B2). These changes were associated with a marked suppression of the two highest frequency components HF and VHF.

To confirm that ME modify the oscillatory frequencies correlated to the endothelial activity, we inhibited ULF or VLF frequency components by local administration of L-NIO or charybdotoxin plus apamin, respectively. We observed that L-NIO administration dramatically decreased the VLF frequency component after ME. Charybdotoxin plus apamin administration, abolished the ULF frequency component after ME.

Based on the observed effects of L-NIO, charybdotoxin, and apamin, our data suggest that ME modulates pial arterioles through the vasodilatory effects of nitric oxide and EDHF acting within the vascular wall.

ME is effective in affecting for long time the mechanisms involved in the regulation of pial arteriolar tone, possibly facilitating the perfusion of cerebral tissue through a modulation of the rhythmic arteriolar diameter changes.

Up to day, the mechanisms whereby ME is followed by a prolonged reduction of blood pressure and heart rate is unclear. As previously reported, the cardiovascular effects caused by ME were abolished by bilateral trigeminal section (Lapi et al., 2013) indicating a role of the trigeminal nerve as the afferent limb. These results are in agreement with the studies by Kumada et al. (1977) and Schaller (2004, 2007), who observed, respectively, in the anesthetized rabbit and in the human during neurosurgical operations, that trigeminal nerve stimulations were followed by (albeit brief) hypotensive and bradycardic responses. From this perspective, ME-induced hypotension may fall into the category of so-called trigemino-cardiac reflexes (TCR) that were extensively reviewed by Schaller (2007). TCR has been proposed to represent the expression of a neuroprotective central neurogenic reflex leading to rapid cerebrovascular vasodilatation in response to facial and nasal mucosal stimulation such as during diving, and that may be also of potential relevance in brain injury states (Schaller, 2004).

In conclusion, for the first time we observed that ME repetition markedly prolongs the already long-lasting decreasing effect on blood pressure and heart rate induced by a single ME in the rat. In addition, repeated ME are followed not only by a protracted dilation of pial arterioles, but also by a change in regulation of their diameters, as indicated by a shift toward lower levels of their frequency components. The mechanisms underlying these effects need to be clarified and further experiments are in progress.

This modulation of the vascular tone produced by the repeated ME determined a redistribution of cerebral blood flow supply. One may speculate that if the observed hemodynamic effects of repeated ME in the rat could be reproduced in the humans. This requires to be confirmed in dedicated studies, and eventually could suggest the development of suitable non-invasive devices which might find a use in the treatment of arterial hypertension and impaired cerebral arteriolar dilation.

## Author contributions

Conceived and designed the experiments: DL, AC, RS. Performed the experiments: DL, MV. Analyzed the data: RS. Contributed reagents/materials/analysis tools: DL, CD, RS. Wrote the paper: DL, RS. Contributed to the editing and revising of the manuscript: DL, CD, SG, EF, AC, RS.

### Conflict of interest statement

The authors declare that the research was conducted in the absence of any commercial or financial relationships that could be construed as a potential conflict of interest.
